# Predictive Factors for the Complications of Dengue Fever in Children: A Retrospective Analysis

**DOI:** 10.7759/cureus.33027

**Published:** 2022-12-27

**Authors:** Nachappa Sivanesan Uthraraj, Laya Manasa Sriraam, Meghanaprakash Hiriyur Prakash, Manoj Kumar, Uthraraj Palanisamy, Kannaki Uthraraj Chettiakkapalayam Venkatachalam

**Affiliations:** 1 Trauma and Orthopaedics, William Harvey Hospital, Ashford, GBR; 2 Otolaryngology, Kanchi Kamak, Chennai, IND; 3 Cardiology, Banner - University Medical Center Phoenix, Phoenix, USA; 4 Otolaryngology - Head and Neck Surgery, Manoj ENT Speciality Hospital, Trichy, IND; 5 Otolaryngology - Head and Neck Surgery, Dhanalakshmi Srinivasan Medical College, Trichy, IND; 6 Pediatrics, Sivameds Multispeciality Hospital, Coimbatore, IND; 7 Pediatrics and Child Health, Thamarai Health Care, Coimbatore, IND; 8 Pediatric Medicine, SIMFFER, Coimbatore, IND; 9 Reproductive Genetics, SIMFFER Foundation, Thamarai Health Care, Coimbatore, IND; 10 Reproductive Medicine, The Fertility Center, Kovai Medical Center and Hospital, Coimbatore, IND

**Keywords:** haemoconcentration, tropical infectious diseases, dengue shock syndrome (dss), dengue hemorrhagic fever (dhf), dengue fever (df)

## Abstract

Background and objective

Dengue fever (DF) and its complications - dengue hemorrhagic fever (DHF) and dengue shock syndrome (DSS) - are major public health problems in Southeast Asia. Predicting the development of DHF and DSS using hematological parameters and ultrasonic signs of vascular leakage will help in reducing morbidity and mortality associated with these diseases. Hence, this study aimed to test the association of platelets and packed cell volume (PCV) on day one (D1) of admission with gallbladder wall thickness (GWT) and ascites, which herald the onset of DHF and DSS.

Methods

The electronic health records of 52 pediatric patients admitted during a mini-outbreak were analyzed to assess platelets and PCV on D1, laboratory and ultrasonography findings, and outcomes. Correlations between D1 hematological parameters and GWT and ascites were tested.

Results

There was a positive correlation between GWT of more than 5 mm and ascites. However, there was no significant correlation of platelets and PCV on D1 with either GWT or ascites and consequently DHF or DSS. All the patients responded to fluid, blood, and supportive therapy. There were no mortalities.

Conclusion

Patients who develop GWT after DF are at an increased risk of developing ascites that deteriorate to DHF and DSS. D1 platelets and PCV are not reliable indicators for predicting the progression or worsening of the disease in the pediatric population.

## Introduction

Dengue fever (DF) is an arboviral infection caused by the dengue virus that belongs to the Flavivirus family. The Aedes mosquito is the vector. It is a public health concern endemic to many countries, particularly in Southeast Asia [1,2]. In fact, Southeast Asia accounts for more than three-fourths of the total cases of DF. Its prevalence has increased steeply over the past three decades with a 30-fold increase internationally in the past 50 years [3-5]. DENV-1-4 are the viral serotypes responsible for the disease. The serotyping of the dengue virus is done by sequence analysis of the C-prM junction of the viral genome [6-8]. Among the serotypes, DENV-2 is the most common [9].

The incubation period for the virus in vivo is about four to eight days. While the majority of infections are self-limiting, plasma leakage due to increased capillary permeability leads to the hemorrhagic variants of the disease: dengue hemorrhagic fever (DHF) and dengue shock syndrome (DSS). DHF mainly affects children under the age of 15 years [1,10].

The clinical manifestations of DF are mainly fever with myalgia. Infection due to a single serotype or, more commonly, cross-infection with a secondary serotype is commonly seen in the pediatric population, leading to DHF characterized by thrombocytopenia and hemoconcentration [increased packed cell volume (PCV)] [11-13]. Plasma leakage manifests as hemoconcentration, pleural effusion, ascites, hypalbuminemia, and hypoproteinemia [12]. The end stage of this spectrum is DSS characterized by circulatory shock with a high mortality rate [14]. DENV-2 serotype patients cross-infected with DENV-4 have a DHF incidence rate of 20% [9].

Hematological investigations can help in diagnosing the disease early and reduce mortality associated with DHF and DSS. Thrombocytopenia is the most common abnormality (68.46%), and the detection of the non-structural protein 1 (NS1) antigen, immunoglobulin G and M in the later stages by enzyme-linked immunosorbent assay (ELISA) and reverse-transcriptase polymerase chain reaction (RT-PCR) are the primary modalities of laboratory diagnosis [1,15].

Gallbladder wall thickness (GWT) is a quantifiable ultrasound sign to establish plasma leakage in patients with DF. GWT between 3-5 mm is associated with more severe cases of dengue [16].

The identification of risk factors for the development of DHF and DSS can help in reducing morbidity and mortality in low- and middle-income countries. Prediction of the onset of complications helps to better treat and escalate care [17]. There is scarce literature on the association between the hematological parameters on admission and the development of complications (DHF and DSS) in the pediatric population. In light of this, the aim of this study is to analyze the correlation of day-one (D1) platelets and PCV to the development of complications exclusively in the pediatric population.

## Materials and methods

This was a retrospective study conducted at a district hospital in India during a dengue virus mini-outbreak over a period of three months. Informed consent was obtained from the parents/guardians of the patients for the purpose of this study. This project was approved by Manoj ENT Speciality - Institutional Ethics Committee (MES-IEC) (approval no: DGBPC-CPS-03 version 0). Patients who presented with signs and symptoms of DF and underwent sera testing for the dengue virus NS1 antigen by ELISA and seropositive patients were admitted. Some patients also underwent testing for IgM and IgG antibodies. The inclusion criteria for the study were as follows: any child below the age of 15 years with a fever for two or more days and a seropositive test for the NS1 antigen. The exclusion criteria were as follows: patients who were non-symptomatic, did not test positive for the NS1 antigen, were above the age of 15 years, had a fever for less than two days duration, or if the parents/guardians declined consent for their children/wards to participate in the study.

On admission, patients were monitored for vitals fourth-hourly; an intake/output chart was maintained with an eighth-hourly consultant review. Complete hemograms including the PCV and platelets were done daily, and ultrasound examinations were done at regular intervals. Tests for IgG and IgM antibodies were done at regular intervals as required. Liver function and renal function tests were done if there was a clinical indication. Abdominal ultrasound was performed by a consultant radiologist using the GE Volusan E8 machine (General Electric Company, Boston, MA). GWT (longitudinal and transverse axial measurements) was recorded. The presence of third-space fluid in the abdomen was screened by scanning the abdominal cavity (ascites), perirenal and pleural spaces along with the liver, kidney, and bowel wall for edema. The presence of free fluid was quantified by the vertical height of the fluid column in the supine position. The scans were done on the day of admission and if the fever persisted or if there was worsening of clinical signs or laboratory parameters. Patients with a GWT of more than 3 mm were managed with fluid therapy, irrespective of their temperature. Patients were discharged when they were asymptomatic with normal platelet counts, PCV, and repeat normal ultrasound scans. There was no mortality in our study.

The data were analyzed using Microsoft Excel (Microsoft Corporation, Redmond, WA) and IBM SPSS Statistics version 28 (IBM Corp., Armonk, NY). The mean and standard deviation (SD) were calculated for age as well as D1 platelets and PCV as continuous variables. The correlation of the variables - D1 platelets, PCV, plasma leak (ascites), and GWT - were tested using Spearman’s test. A p-value <0.05 was considered statistically significant.

## Results

The total number of patients was 52, with a mean age of 9.17 years (SD=4.04). The average number of days of fever on admission was 3.19 days (SD=0.63). The mean platelet count was 162,000/cu.mm (SD=52.8). The mean hematocrit (PCV) was 38.6% (SD=3.69) (Table 1). Of note, 92.3% of the patients had D1 platelets of less than 100,000/cu.mm and 67.3% of the patients had D1 PCV greater than 40%. Plasma leakage, as measured by the fluid in the abdomen (ascites) on ultrasonography was 0.385 cm (SD=0.491) on average; 61.5% of the patients had evidence of plasma leak. The mean GWT on ultrasonography was 0.308 cm (SD=0.47). Of note, 69.2% of the patients had GWT of more than 5 mm. On correlational analysis, with the D1 platelet count and PCV as continuous variables, there was no significant correlation of D1 platelet count or PCV levels with the development of plasma leak or GWT greater than 5 mm. However, there was a significant positive correlation between plasma leak and GWT of more than 5 mm (Table 2).

**Table 1:** Demographics, hospital course, and hematological and radiological parameters

Variable	Mean	Standard deviation
Age	9.17 years	4.04
Number of days of admission	3.19	0.63
Platelet count	162,000/cu.mm	52.8
Packed cell volume (PCV)	38.6%	3.69
Ascitic fluid	0.385 cm	0.491
Gall bladder wall thickness (GWT)	0.308 mm	0.47

**Table 2:** Correlational analysis involving day 1 platelets, packed cell volume, gall bladder wall thickness, and ascites

	Statistical test/factor	Day 1 platelets	Day 1 packed cell volume	Ascites	Gall bladder wall thickness >5 mm
Day 1 platelets	Spearman’s rho	_	_	_	_
	P-value	_	_	_	_
Day 1 packed cell volume	Spearman’s rho	-0.144	_	_	_
	P-value	0.309	_	_	_
Ascites	Spearman’s rho	-0.190	0.063	_	_
	P-value	0.178	0.656	_	_
Gall bladder wall thickness >5 mm	Spearman’s rho	-0.083	-0.096	0.758	_
	P-value	0.557	0.499	<0.001	_

## Discussion

This study was a retrospective analysis among the pediatric population aged between 1-15 years, admitted with fever and a positive dengue antigen (NS1) test. The objective was to determine if the D1 platelet count or PCV could predict the onset of a vascular leak (plasma leak) or GWT, heralding the onset of DHF and DSS. Our findings showed that there was no correlation of D1 platelet count and PCV with GWT or vascular leak. However, there was a significant positive correlation between GWT >5 mm and vascular leak, irrespective of the D1 hematological parameters. This is the first study to test these correlations in the pediatric population.

Vijay et al. have retrospectively reported on a series of dengue patients, from the same state as ours in India, but in an urban demographic and in an adult population. Of note, 77% of the patients had a platelet count of less than 100,000/cu.mm; 15.3% had evidence of ascites due to plasma leak, and 47.6% had a hematocrit of greater than 40% [17]. In our series, 92.3% had a platelet count of less than 100,000/cu.mm on D1, 61.5% of patients had evidence of plasma leak, and 67.3% had a hematocrit of greater than 40% on D1. In the series by the above-mentioned author, it was unclear if the hematological parameters were measured on D1 or if it was the average for the duration of the admission. This and the fact that the study was in an adult population in comparison to our pediatric cohort could explain the higher rates. It could also be inferred from comparing the two studies that the percentage of patients who progress to DHF and DSS is higher in the pediatric population.

DENV-2 is the most common subtype in India and Pakistan, with one outbreak in Pakistan, as an outlier, caused mainly due to the DENV-4 subtype [6,7,9]. The DENV-2 serotype has the highest association with severe dengue, DSS, and secondary cross-infections [4,12]. We did not perform the serotype subgroup analysis in our study population.

Outbreaks in the northern part of India and Pakistan involving the adult and pediatric population did not result in any mortality [7,9]. A systematic review and meta-analysis estimated the mortality to be at 1.3% [4]. There was no mortality in our pediatric series despite the fact that patients developed hemorrhagic complications.

A systematic review and meta-analysis by Huy et al. concluded that thrombocytopenia and plasma leakage (hemoconcentration, pleural effusion, and ascites) increased the risk of DSS [12]. Ascites was identified as a predictor for mortality in another systematic review and meta-analyses [4]. We could not find a correlation of hemoconcentration and thrombocytopenia (D1) with DSS or DHF. Selecting D1 hematological parameters as variables could have undermined the association with severe DF in our analysis.

Data from an outbreak in the Punjab province of Pakistan showed a 60% incidence of DHF and DSS. Thrombocytopenia, leukopenia, and deranged LFTs were linked to the severe forms of the disease. Another outbreak in the same province was reported by the same authors with children under the age of 15 years accounting for more than half of the cohort. There was no mortality and all the patients responded well to fluids, blood products, and supportive therapy [7]. The incidence rates of DHF and DSS were very similar in our study. The pediatric population responds well to therapies, as evidenced by our study and that of others. Some other studies from the Indian subcontinent have reported a higher predilection for dengue viral infection in children less than 15 years of age [18-20]. Since our study was exclusively done in a pediatric population, a comparison could not be made to comment on the propensity.

Arshad et al. have reported on a cohort of adult dengue patients with a mean platelet count of 145,220/cu.mm. Both antigen and antibody tests were done for all the patients with 85.5% testing positive for the NS1 antigen and 34% testing positive for IgG or IgM. Those with dengue-specific antibodies were less likely to bleed; 31.2% tested positive for both antibodies. Thrombocytopenia was associated with bleeding, and blood product transfusion increased the survival chances in these patients [13]. In our series, 5.7% tested positive for IgM, 15.3% for IgG, and 3.8% for both IgG and IgM antibodies. Since the numbers were low, we did not test the association of antibody-positive patients with bleeding or the severity of the disease.

Adil et al. drew an association of the evidence of fluid leak manifesting as gall bladder wall thickness, ascites, and low platelet count with DHF and DSS. Interestingly, there was no correlation between DHF/DSS and increased hematocrit (PCV), which is another indicator of plasma leakage. The critical value for determining GWT was 3 mm from their results [16]. Their methodology was very similar to ours, although we dealt with a pediatric population. We could partially validate the association of plasma leakage (GWT) with severe disease. We tested correlations with a GWT of 5 mm in children. However, we could not establish a predictive association between thrombocytopenia on D1 and DHF/DSS.

Vascular leak and thrombocytopenia are seen after the febrile episode, and predicting its occurrence is important for implementing pre-emptive measures [17]. This acquires even more importance in a low-resource setting like our study center for pre-emptive planning of escalation of care. There is also a high risk of vulnerable patients being inadvertently discharged. Predictive modeling would help physicians identify the subset of patients who would need inpatient care, after the resolution of the initial febrile episode.

The major strength of this study is that it is one of the few to analyze the association between D1 hematological parameters and complications (DHF/DSS), specifically in a pediatric population. This will help pediatricians in low-resource settings to devise more robust treatment plans including escalation of care, thereby reducing morbidity and mortality. The routine use of ultrasonography for DF admissions in the pediatric population and daily laboratory assays have been validated through this series.

This study has a few limitations. Primarily, it was conducted at a single center over a brief period of time. We did not engage in a comparative analysis with the adult population from the same region to see if there are differences in terms of disease progression. Serotype classification could have been done to compare the results with other similar studies. Moreover, there could be other confounding factors leading to DHF or DSS, which were not analyzed in this study.

## Conclusions

Based on our findings, in the pediatric population, hematological parameters such as D1 platelet count and PCV are not reliable predictors of DHF or DSS. GWT heralds the onset of severe dengue. Daily hematological assays and serial ultrasound scans should be instituted as part of the protocol to manage hospitalized dengue patients. Future research in this field could expand on our methods to include the white cell count, aspartate transaminase, alanine transaminase, and albumin levels as predictive factors for DSS or DHF.

## References

[1] Guzman MG, Harris E (2015). Dengue. Lancet.

[2] Uno N, Ross TM (2018). Dengue virus and the host innate immune response. Emerg Microbes Infect.

[3] R R, V. (2012 (2022). WHO: global strategy for dengue prevention and control, 2012-2020. https://www.who.int/publications/i/item/9789241504034.

[4] Guo C, Zhou Z, Wen Z (2017). Global epidemiology of dengue outbreaks in 1990-2015: a systematic review and meta-analysis. Front Cell Infect Microbiol.

[5] Murugesan A, Manoharan M (2019). Dengue virus. Emerging and Reemerging Viral Pathogens.

[6] Fatima Z, Idrees M, Bajwa MA (2011). Serotype and genotype analysis of dengue virus by sequencing followed by phylogenetic analysis using samples from three mini outbreaks-2007-2009 in Pakistan. BMC Microbiol.

[7] Humayoun MA, Waseem T, Jawa AA, Hashmi MS, Akram J (2010). Multiple dengue serotypes and high frequency of dengue hemorrhagic fever at two tertiary care hospitals in Lahore during the 2008 dengue virus outbreak in Punjab, Pakistan. Int J Infect Dis.

[8] Mahmood N, Rana MY, Qureshi Z, Mujtaba G, Shaukat U (2012). Prevalence and molecular characterization of dengue viruses serotypes in 2010 epidemic. Am J Med Sci.

[9] Gupta A, Rijhwani P, Pahadia MR (2021). Prevalence of dengue serotypes and its correlation with the laboratory profile at a tertiary care hospital in Northwestern India. Cureus.

[10] Gubler DJ (1998). Dengue and dengue hemorrhagic fever. Clin Microbiol Rev.

[11] Teoh BT, Sam SS, Tan KK (2013). Dengue virus type 1 clade replacement in recurring homotypic outbreaks. BMC Evol Biol.

[12] Huy NT, Van Giang T, Thuy DH, Kikuchi M, Hien TT, Zamora J, Hirayama K (2013). Factors associated with dengue shock syndrome: a systematic review and meta-analysis. PLoS Negl Trop Dis.

[13] Arshad H, Bashir M, Mushtaq US (2022). Clinical characteristics and symptomatology associated with dengue fever. Cureus.

[14] Soo KM, Khalid B, Ching SM, Chee HY (2016). Meta-analysis of dengue severity during infection by different dengue virus serotypes in primary and secondary infections. PLoS One.

[15] Patel K, Patel D, Das VK (2013). Hematological parameters and its utility in dengue fever: a prospective study. Int J Sci Res.

[16] Adil B, Rabbani A, Ahmed S, Arshad I Sr, Khalid MA (2020). Gall bladder wall thickening in dengue fever - aid in labelling dengue hemorrhagic fever and a marker of severity. Cureus.

[17] Vijay J, Anuradha N, Anbalagan VP (2022). Clinical presentation and platelet profile of dengue fever: a retrospective study. Cureus.

[18] Qureshi JA, Notta NJ, Salahuddin N, Zaman V, Khan JA (1997). An epidemic of dengue fever in Karachi--associated clinical manifestations. J Pak Med Assoc.

[19] Singh NP, Jhamb R, Agarwal SK (2005). The 2003 outbreak of dengue fever in Delhi, India. Southeast Asian J Trop Med Public Health.

[20] Kittigul L, Pitakarnjanakul P, Sujirarat D, Siripanichgon K (2007). The differences of clinical manifestations and laboratory findings in children and adults with dengue virus infection. J Clin Virol.

